# MicroRNA-124 suppresses the migration and invasion of osteosarcoma cells via targeting ROR2-mediated non-canonical Wnt signaling

**DOI:** 10.3892/or.2021.8107

**Published:** 2021-06-07

**Authors:** Can Zhang, Yihe Hu, Jun Wan, Hongbo He

Oncol Rep 34: 2195-2201, 2015; DOI: 10.3892/or.2015.4186

Following the publication of this paper, it was drawn to the Editors attention by a concerned reader that certain of the cell Transwell assay data in the article (featured in Figs. 2C and 5B) were strikingly similar to data appearing in different form in other articles by different authors, which were either already under consideration for publication or had already been published elsewhere at the time of the present articles submission.

Owing to the fact that the contentious data in the above article were either already under consideration for publication, or had already been published elsewhere, prior to its submission to *Oncology Reports*, the Editor has decided that this paper should be retracted from the Journal. After having been in contact with the authors, they agreed with the decision to retract the paper. The Editor apologizes to the readership for any inconvenience caused.

